# Epidemiological Analysis of HPV Infection in Zhangjiagang, Southern Jiangsu Province of China: A Cross-Sectional Study

**DOI:** 10.1155/ijm/5576260

**Published:** 2025-04-19

**Authors:** Qin Rui, Xiaojue Zhu, Guoxin Xu

**Affiliations:** ^1^Department of Center of Clinical Laboratory, The Fourth Affiliated Hospital of Soochow University, Suzhou Dushu Lake Hospital, Medical Center of Soochow University, Suzhou, China; ^2^Department of Laboratory, The Affiliated Zhangjiagang Hospital of Soochow University, Suzhou, China

**Keywords:** cervical cancer, genotypes, high-risk HPV, human papillomavirus, prevalence

## Abstract

An examination of the fluctuating rates of the human papillomavirus (HPV) prevalence and the allocation of various genotypes could assist in the implementation of targeted strategies for the prevention and treatment of cervical cancer. The present study was aimed at evaluating the prevalence of HPV and genotype distribution among female populations in Zhangjiagang, Jiangsu Province, China. A total of 62,036 patients from the Affiliated Zhangjiagang Hospital of Soochow University provided clinical specimens between January 2022 and August 2023, and 12,144 women were infected with HPV, with an overall positive rate of 19.58%. The most prevalent HR-HPV types were HPV52 (4.14%), HPV16 (2.44%), HPV53 (2.37%), HPV58 (2.37%), and HPV56 (1.50%). In the low-risk group, the highest infection rate was HPV81 (1.76%). The prevalence of HPV infection showed a “U”-shaped curve. In addition to the adolescent group, other age groups are mainly single infection. And the gynecology clinic group had much higher rates of HPV positivity compared to the physical examination group (*p* < 0.001). Our study suggests that HPV screening and vaccination in young women are highly necessary, and 9-valent HPV vaccine is recommended for regular immunization in Zhangjiagang city.

## 1. Introduction

Human papillomavirus (HPV) infection and its associated diseases continue to be a significant global public health issue [1]. Cervical cancer caused by HPV is the fourth most common cancer in women globally [2]. In China, the incidence of cervical cancer ranks sixth among women, and the number of deaths ranks seventh. In 2020 alone, China reported around 109,741 new cases of cervical cancer, accounting for 18.2% of all cases, and documented 60,590 fatalities, or 17.3% of all cervical cancer–related deaths [3]. In addition, the incidence of oropharyngeal cancer in men caused by HPV infection is increasing year by year in developed countries. The percentage of OPSCCs that are HPV+ was reported in 2021 to be 33%, globally [4]. HPV is a minuscule, nonenveloped virus with double-stranded DNA, and it exhibits a wide range of genotypes, with over 450 genotypes having been identified and analyzed thus far. The World Health Organization (WHO) categorizes it as high or low risk depending on its L1 gene sequence and its associated risk of cancer [5, 6].

In the absence of clinical therapy, most HPV infections are short-lived and self-limiting, but persistent high-risk HPV (HR-HPV) infection is a prerequisite for developing cervical cancer. Therefore, early and regular screening for HPV infection is of great significance for the prevention of cervical cancer [7]. Fortunately, cervical cancer is now one of the most preventable and treatable malignancies in the world due to the development of vaccines [8]. Being vaccinated is the best way to prevent HPV infection, cervical cancer, and other HPV-related cancers. In November 2020, WHO proposed a global strategy to expedite the elimination of cervical cancer by raising HPV vaccination coverage to 90%, screening coverage for cervical lesions to 70%, and access to cervical cancer treatment to 90% by 2030 [9]. Screening can detect cervical precancers that can be treated before they develop into cancer. HPV vaccines should be given to all girls aged 9–14 years before they become sexually active [8, 9].

The HPV vaccine is currently not part of China's national vaccination program, and the country promotes experimental trials in places where conditions allow. Due to the fact that different types of HPV have different carcinogenicities and because of the differences in climate, age, living habits, and population distribution, the primary types of HPV infected by people in different regions are also different [10]. As a result, investigating the state of HPV infection in a given location is vital to leading the creation of prevention and control strategies in the region. The purpose of this study was to look into the HPV infection rate and type distribution in women of all ages in Zhangjiagang and to provide a reference for the development of clinical preventive and treatment measures. The following is the report.

## 2. Materials and Methods

### 2.1. Study Population

In this study, the data of 62,036 females from the Affiliated Zhangjiagang Hospital of Soochow University between January 2021 and August 2023 were retrospectively analyzed. Two classification methods were used. (1) The participants were divided into six age groups: the G1 group aged < 18 years, the G2 group aged ≥ 18 years but < 30 years, the G3 group aged ≥ 30 years but < 40 years, the G4 group aged ≥ 40 years but < 50 years, the G5 group aged ≥ 50 years but < 60 years, and the G6 group aged ≥ 60 years. (2) All patients were separated into two groups: those who had a physical examination (PEG) and those who had a gynecological clinic (GCG).

Women were excluded from this research if they had been diagnosed with cervical cancer, were pregnant at the time of enrollment, lacked age information, had a hysterectomy, or were immunocompromised. To preserve the research participants' privacy, identifiable information was removed before analysis. The study obtained clearance from the ethical committee of the specified hospitals, and all participants provided signed informed consent upon recruitment.

### 2.2. Sample Collection and DNA Extraction

Clinicians extracted the exfoliated cells with a speculum, and a cervical brush was turned clockwise four to five times to get enough cervical epithelial cells. The cells were placed in sterile sample containers with cell preservation solution and kept at 4°C. DNA was isolated from exfoliate cells using a DNA extraction kit (Yanengbio, Shenzhen, China) according to the manufacturer's instructions. After mixing, the cell-containing preservation solution was transferred into a 1.5-mL centrifuge tube and centrifuged at 13,000 rpm for 10 min. The supernatant was discarded, and 100 *μ*L nucleic acid extract was added to suspend the pellet. Finally, the mixture was heated in a metallic bath for 10 min and then centrifuged at 13,000 rpm for 10 min at 4°C, with the supernatant saved for later use.

### 2.3. HPV DNA Genotype Testing

HPV DNA genotype testing was performed using the HPV genotyping test kit in accordance with the manufacturers' instructions (Yanengbio, Shenzhen, China), which can detect 17 HR-HPV genotypes (16, 18, 31, 33, 35, 39, 45, 51, 52, 53, 56, 58, 59, 66, 68, 73, and 82) and 6 low-risk HPV genotypes (6, 11, 42, 43, 81, and 83). HPV DNA was amplified using a GE961-B instrument (Bio-Gener, China) following the manufacturer's guidelines as follows: 50°C for 15 min, 95°C for 10 min, 94°C for 30 s, 42°C for 90 s, 72°C for 30 s (40 cycles), and finally 72°C for 5 min. Then, it was hybridized with a hybridizer (YN-H96, Yanengbio, China). Quality control was conducted throughout the experiments, including DNA extraction and amplification by positive and negative controls.

### 2.4. Statistical Analysis

SPSS 21.0 (IBM, Armonk, New York, United States) was utilized for data analysis. Single, double, and multiple HPV infections were defined as infections with one, two, or more than two genotypes of HPV. We used SPSS 21.0 to figure out the HPV prevalence in different groups and the corresponding 95% confidence intervals (95% CI). The chi-square test was then used to compare the infection rates based on disease group. Categorical variables were utilized to examine changes in the HR-HPV prevalence across age groups when comparing various age groups. For comparisons among different age groups, categorical variables were used to assess changes in the HR-HPV prevalence across the age groups. Statistical significance was identified as *p*-values < 0.05.

## 3. Results

### 3.1. The Overall Prevalence of HPV Infection and Age Groups

**Figure 1:**
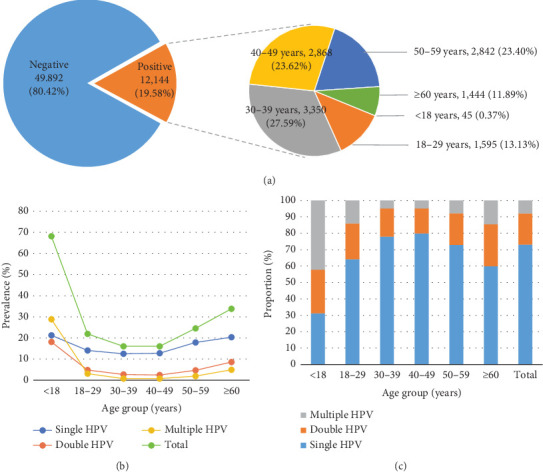
Overall analysis of HPV among women in different age groups. (a) The overall infection rate of HPV among women in different age groups. (b) The prevalence of single-type or multitype HPV infection rate among women in different age groups. (c) The proportion of single-type or multitype HPV infection rate among different age women.

In this study, 62,036 women from November 2021 to August 2023 from the Affiliated Zhangjiagang Hospital of Soochow University met the selection criteria (Figure 1). The age of the participants ranged from 13 to 98 years. 19.58% (12,144/62,036, 95% CI: 19.26%–19.89%) of participants were tested positive for one or more HPV genotypes (Figure 1). The participants were divided into six groups according to age. The prevalence of HPV infection in each age group was calculated, as shown in Table 1 and Figure 1. The HPV infection rate calculated for each age group ranged from 16.01% to 68.18%. The highest HPV infection rate was observed in G1 (68.18%), followed by G6 (33.83%), G5 (24.48%), G2 (21.97%), G3 (16.01%), and G4 (16.01%). The prevalence initially decreased from the peak observed among women under 18 years old until the 40–49 age group, after which it showed an upward trend from the 40–49 age group to the > 60 age group (Table 1, Figure 1a).

### 3.2. Distribution of Single, Double, and Multiple HPV Infections in Different Ages

Among the total cases, single HPV infections were the most common and accounted for 73.16% (8884/12,144) of the positive cases, while double infections accounted for 18.80% (2283/12,144) of the positive cases. Multiple HPV infections accounted for 8.04% (977/12,144) of the positive cases (Table 2).

The proportions of single, double, and multiple infections in each age group were further analyzed (Figure 1c, Table 3). The prevalence of multiple infections was also the lowest in all age groups except for women younger than 18. In this group, the proportion of single infection in this age group was 31.11%, being the lowest in all age groups. It reveals that HPV infection is often a combination of two or more subtypes among minors (Figure 1c). But due to the small number of people screened in this group, this set of data is for reference only. Starting from the age group of 18–29, whether it is single infection or coinfection, the infection trend was consistent with the overall infection trend (Figure 1b). And except for the minors, the proportion of single infection is more than 60%, even up to 80% among the 40–49 age group (Figure 1b, Table 3).

### 3.3. Infection and Distribution Characteristics of Different HPV Genotypes

**Figure 2:**
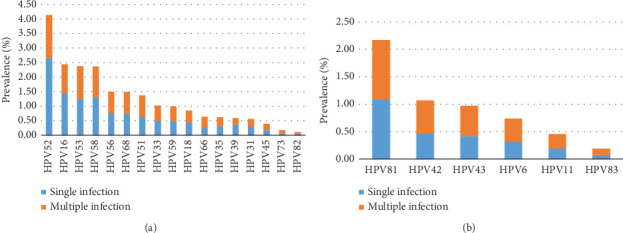
Distribution of different HPV genotypes among samples. (a) The prevalence of HR-HPV among samples. (b) The prevalence of LR-HPV among samples.

Table 3 and Figure 2 show the prevalence of different HPV genotypes as well as the proportion of single or multiple infections for each subtype. It was shown that the top five subtypes of HR-HPV infection are HPV52, HPV16, HPV53, HPV58, and HPV56, with the top subtype HPV52, being roughly 1.7 times more common than the second subtype HPV16. In the LR-HPV group, the highest infection rate was HPV81, which was more than double the incidence of the next subtype, HPV42 (Table 4). We also discovered that in the HR-HPV group, there was little difference in the incidence of single or coinfection for each type, except for subtype HPV52, of which single infection was predominant, accounting for nearly two-thirds (Table 4, Figure 2a). Interestingly, coinfection accounted more for each subtype among LR-HPV group (Table 4, Figure 2b).

### 3.4. HPV Genotype Distribution Across Different Age Groups

**Figure 3:**
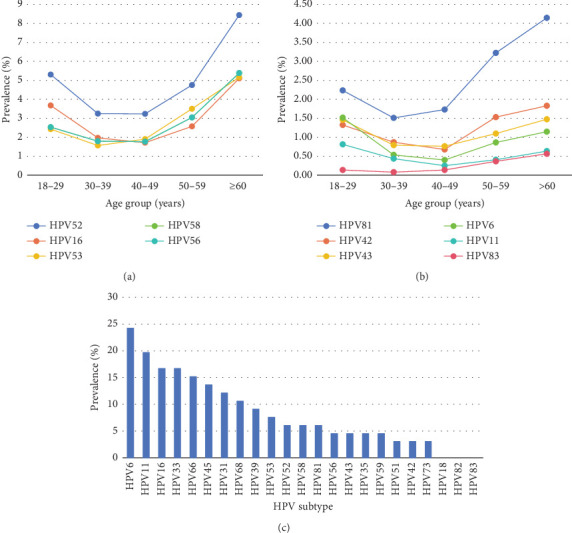
Distribution of different HPV genotypes among women in different age groups. (a) The prevalence of the top five HR-HPV in different adult age groups. (b) The prevalence of LR-HPV infection in different adult age groups. (c) The distribution of HPV genotypes among minors.

The frequency of HPV genotype infections in different age groups is shown in Table 5. Due to the small sample size under 18 years old, we made a separate subtype distribution analysis for this group (Figure 3c). The distribution of the top five HR-HPV subtypes and LR-HPV in other age groups is shown in Figure 3a,b. Similar to the total prevalence of HPV infection, any LR-HPV infections and HR-HPV infections also exhibited these two peaks in the same age groups. Unlike adults, the top five infection genotypes among minors are HPV6, HPV11, HPV16, HPV52, and HPV58, which indicated that in the adolescent HPV infection group, HPV6 and HPV11 caused condyloma acuminatum to account for the majority (Figure 3c).

### 3.5. Prevalence and Distribution of HPV Infection in GCG and PEG

**Figure 4:**
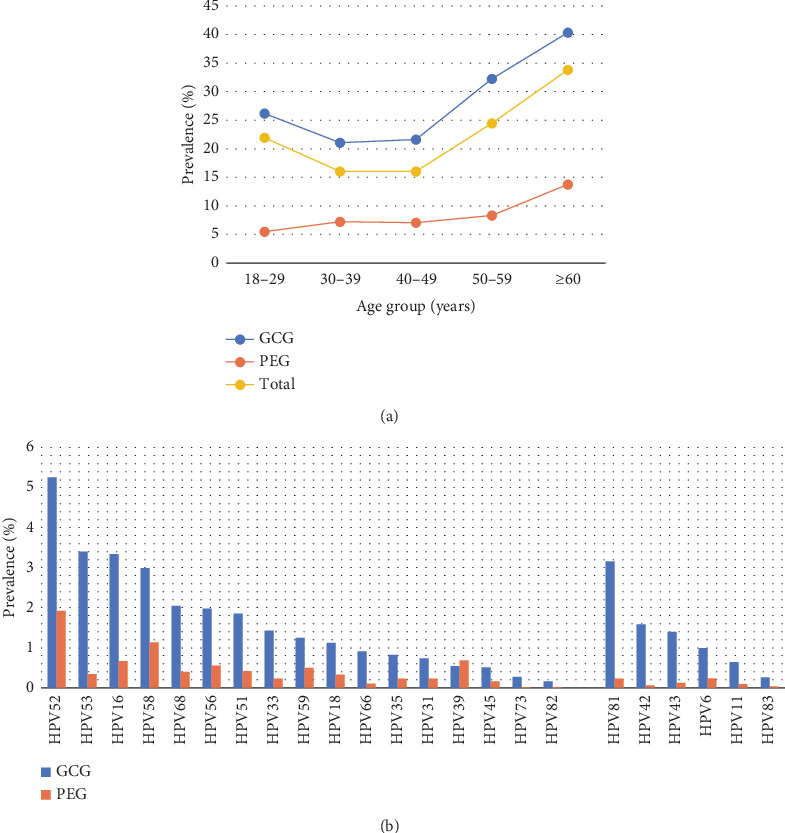
Infection rate analysis of HPV grouped by participant type. (a) The prevalence of HPV among different age women in GCG and PEG groups. (b) The genotype distribution of HPV in GCG and PEG groups.

According to the attributes of the screening subjects, they were divided into GCG, (*n* = 41,174) and PEG (*n* = 20,862). As shown in Table 6, the total HPV infection rate in the GCG was 25.64% (95% CI: 25.22%–26.06%), which was significantly higher than that in the PEG group (7.60%, 95% CI: 7.24%–7.96%, *p* < 0.001). The HPV genotype distribution across different age groups of GCG and PEG is shown in Table 6 and Figure 4a. It reveals that the HPV infection rate of the PEG subjects showed an increasing trend with age. The distribution of HR-HPV subtype infection rates for GCG and PEG is shown in Figure 4b and Table 7. The top five HR-HPV subtypes in the GCG group were HPV52, HPV53, HPV16, HPV58, and HPV68. For the PEG, the most common HR-HPV subtypes were HPV52, HPV58, HPV16, HPV56, and HPV59, whereas the highest LR-HPV infection types of the GCG and PEG were HPV81 and HPV6, respectively (Figure 4b, Table 7).

## 4. Discussion

In this study, we established that the general HPV infection rate among women in Zhangjiagang was 19.58%, with 25.64% in GCG and 7.60% in PEG, respectively. Our results are consistent with a previous study on HPV infection rates in Jiangsu by Zhang et al. [11] and another study carried out in Yangzhou (23.56%) [12]. Zhang et al. selected three cities, Xuzhou, Nanjing, and Suzhou, as representatives and analyzed the overall HPV infection rates, which were 26.92%, 24.04%, 28.79%, and 28.72%, respectively, in these three cities [11, 13]. In another recent article analyzing HPV infection rates in Suzhou by Wang et al., the HR-HPV prevalence was 10.20%. Since the population included in that study are women aged 35–64, who are permanent residents of the jurisdiction [14], therefore, the infection rate of PEG in our study is closer to theirs. And compared to one study from Changzhou, which was another city in the southern Jiangsu region, we found that our results were significantly higher than theirs (13.60%) [1]. Considering that Zhangjiagang is a port city, the mobility of migrants and differences in detection methods may be explanations for the discrepancy. When compared with another provinces, the HPV prevalence reported in this paper was higher than that in Fujian (15.13%) [15] and Xinjiang (9.15%) [16], but lower than that in Hunan (20.50%) [17], Shandong (22.64%) [18], and Shanxi Province (22.97%) [19]. Divergences in detection methods might potentially contribute to this phenomenon, alongside cultural background and lifestyle choices [20].

Gaining insight into the distribution of HPV genotypes in particular regions will facilitate the development of more effective protective measures. Previous studies have demonstrated that HPV52 and HPV58 were the more prevalent genotypes in Asia, especially in China, and infection with them may have an association with cervical cancer development [20, 21]. We revealed that the top five prevalent HR-HPV infections were HPV52 (4.14%), HPV16 (2.44%), HPV53 (2.37%), HPV58 (2.37%), and HPV56 (1.50%), which in Wang et al.'s [13] study were HPV52 (2.82%), HPV58 (1.64%), HPV16 (1.46%), HPV68 (0.89%), and HPV51 (0.85%) and in Changzhou study [1] were HPV52 (3.30%), HPV58 (1.91%), HPV53 (1.77%), HPV16 (1.48%), and HPV51 (0.97%). It can be seen that the infection rate of subtypes HPV52, HPV58, and HPV16 ranked in the top five genotypes, but the position order was slightly different. HPV16 and HPV18 have been reported to be the most commonly encountered genotypes worldwide, accounting for up to 70% of cervical cancers [22, 23]. In our study, HPV16 ranked second, whereas HPV18 was only the 10th common HR-HPV genotype. Furthermore, our results revealed that the most prevalent LR-HPV infections were mainly HPV81 and HPV42, which is also different from what has been reported in many other places. It means that bivalent and quadrivalent vaccines, which are effective against HPV16 and HPV18 infections, fail to prevent HPV52 and HPV58 infections. Furthermore, only 9-valent vaccines provided coverage for these two subtypes. Other of the top few genotypes, HPV53, HPV81, and HPV42, are not included in the current available vaccines [8]. These genotypes should potentially be incorporated into future HPV vaccines as a preventive measure against the development of cervical cancer associated with HPV in Zhangjiagang.

This study examined the age-specific distribution of single, dual, and multiple HPV types, as well as their distribution across various subtypes. Aside from the teenage group, the other age groups predominantly experience singular infections. Among the top five high-risk kinds, the prevalence of single infection was somewhat greater than that of multiple infections. It is unknown if numerous infections raise the risk of cervical cancer. According to various studies, multiple infections result in a longer persistence of HPV infection than a single infection, which may contribute to cervical cancer development [24]. On the contrary, several studies found that a single HPV infection was linked to a higher risk of developing cervical cancer than many infections, with competition or balance between different HPV subtypes being one possible pathogenic mechanism [25]. Persistent HR-HPV infection is a prerequisite for cervical cancer, and another crucial step in the development of HPV-related cancer is the integration of the HPV genetic material into the DNA of the host [26]. This procedure entails the suppression of the viral oncogenes E6 and E7 [26, 27]. The oncoprotein E6 has a major role in cell immortalization. It can bind to the tumour suppressor protein p53, which is usually activated in response to DNA damage in normal cells. This binding degrades the protein p53, which prevents the cell from undergoing apoptosis. The oncoprotein E7 also interacts with numerous host proteins, and more specifically, it can bind to the cellular tumour suppressor, pRb. This binding to pRb leads to its degradation, which prevents it from inactivating transcription factors and therefore favouring cell proliferation [27, 28]. Peng et al. found that the presence of E6/E7 mRNA was more prevalent in cases of multiple HPV infections compared to single infection [26]. Additional research is required to investigate the precise mechanism by which single or many HPV infections elevate the likelihood of developing cervical cancer. The examination of dual and multiple HPV infections holds immense importance in providing guidance for the advancement of forthcoming polyvalent HPV vaccines of the second generation.

HPV infection is strongly influenced by age, making it a significant risk factor. In line with most other reports, a bimodal “U” curve for HPV prevalence versus age was also observed in our study. The results showed that HPV infection exhibited a first peak in adolescents under 18 years and a second peak in the > 60 age group.  Figure 3 revealed that the teenage group had a notably elevated infection rate, mostly attributable to the limited sample size. Hence, this group is individually categorized in the subsequent type analysis. The following subtype analysis also showed that both HR-HPV and LR-HPV infections showed this “U” curve pattern. The heightened sexual activity and underdeveloped immune defence mechanisms in young women, along with the physiological and immunological disturbances caused by hormonal changes during the menopausal transition in older women, may account for these two peaks [2, 29]. Interestingly, we can see that the top five HPV infections in the adolescent population, HPV6, HPV11, HPV16, HPV52, and HPV58, are significantly different from those in adults. We acknowledge that the small sample size of adolescents, particularly the limited data from the physical examination population, restricts our ability to calculate the overall HPV infection rate accurately. However, the analysis of HPV type distribution in this group offers important preliminary insights into the prevalence of specific HPV strains among adolescents. We recognize the urgent need for larger scale studies to better understand HPV infection dynamics in adolescents. Our follow-up investigation aims to expand the sample size significantly, to improve our understanding of HPV infection in adolescents, though this will require time due to the challenges, including stigma, lack of awareness, and parental concerns. We plan to collaborate with schools and community organizations to conduct educational campaigns targeting both adolescents and their families. These efforts will emphasize the importance of HPV testing and vaccination in preventing serious health outcomes. In addition, studies showed that the risk of HPV infection in women who had sex for the first time under the age of 16 was about three times that of those who had sex for the first time at age ≥ 21 [30]. Therefore, our study will prioritize 14–18-year-old girls who have had sex, due to their potentially lower awareness of safety precautions compared to adults. Although the adolescent population has a higher infection rate, it is fortunate that these types with high infection rates are all included in the 9-valent vaccine. The results of an 8-year follow-up study of three doses of 9-valent HPV vaccine in girls aged 9–15 years showed that the positive rate of HPV antibodies against vaccine-associated types was more than 90% [31]. Thus, it is crucial for teenagers to receive vaccinations in order to decrease the incidence of primary HPV infection. In addition, based on a study examining HPV vaccination intentions and influencing factors among Chinese male and female college students, it was found that female students possessed superior knowledge and attitudes towards HPV in general. Conversely, male students exhibited a higher perception of susceptibility to HPV infection compared to their female counterparts [32]. Consequently, it is imperative to introduce improved educational programs aimed at augmenting university students' comprehension and viewpoints regarding HPV and HPV vaccine [32].

Since participants in this study included gynecological outpatients and physical examination crowds, we analyzed the distribution of overall HPV positive rates and HPV subtype infection rates in the two groups. The data indicated that the GCG had an HPV infection rate of 25.64%, which was much greater than the 7.6% rate observed in the PEG. Age analysis revealed that, in contrast to the GCG, the PEG exhibited a positive correlation between HPV infection rate and age rather than a distribution following a “U” curve pattern. Furthermore, with the exception of HPV52, the remaining predominant subtypes in the two groups were distinct. Given that gynecological outpatients exhibit specific symptoms, the findings from the physical examination group can be considered somewhat indicative of the screening outcomes for women in society.

This study described the prevalence and characteristics of HPV among women in Zhangjiagang city. There are several limitations of this study. First of all, this study did not analyze the HPV infection rate of men in Zhangjiagang city. Oropharyngeal cancer caused by HPV infection is on the rise in men, especially in developed countries. In both the United Kingdom and the United States, the incidence of oropharyngeal cancer in men has even surpassed that of cervical cancer in women [4]. The most frequently associated risk factor of HPV-positive OPSCC is lifetime number of oral sex partners. Similar to cervical cancer, HPV Type 16 is currently recognized as the leading cause of OPSCC, followed by HPV18 [33, 34]. Second, this study was only a hospital-based survey, including women who visited our hospital and received HPV DNA genotyping from January 2021 to August 2023, which may not represent the general population in Zhangjiagang. Third, this study did not document the personal details of the patients, such as their HPV vaccination status and sexual activities. As a result, it was not possible to determine the specific influence of diverse backgrounds on the prevalence of HPV infection and the distribution of genotypes. Fourth, the association between the distribution of HPV infection genotypes and other cervical abnormalities cannot be established due to the absence of cervical cytological or histological evidence.

## 5. Conclusion

In summary, this investigation unveiled the prevalence and genotype distribution of HPV in Zhangjiagang city, which was basically consistent with the prevalence of HPV in China. Our findings demonstrated a very high prevalence of HPV infection in young women, suggesting that HPV screening and vaccination in young women are highly necessary. Women aged 50 and above who have contracted HR-HPV for a duration exceeding 1 year should undergo screening and get vigilant monitoring. These findings offer essential data for cervical cancer screening and important direction for local governments to facilitate future HPV-targeted immunization efforts.

## Figures and Tables

**Table 1:** The prevalence of HPV among women in different age groups.

**Group**	**Age, years**	**Sample sizes**	**Ratio (%)**	**Positive no**	**Prevalence (95% CI, %)**
G1	< 18	66	0.11	45	68.18 (56.94–79.42)
G2	18~29	7259	12.09	1595	21.97 (21.02–22.93
G3	30~39	20,922	33.21	3350	16.01 (15.51–16.51)
G4	40~49	17,913	28.62	2868	16.01 (15.47–16.55)
G5	50~59	11,608	18.82	2842	24.48 (23.70–25.27)
G6	≥ 60	4268	7.14	1444	33.83 (32.41–35.25)
Total		62,036		12,144	19.58 (19.26–19.89)

**Table 2:** The prevalence of single and multiple HPV infection (*N* = 12,144).

**Genotype of HPV infection**	**Numbers of cases**	**Ratio (%)**	**Prevalence (95% CI, %)**
1 HPV subtype	8884	73.16	14.32 (14.07–14.57)
2 HPV subtype	2283	18.8	3.68 (3.55–3.81)
3 HPV subtype	663	5.46	1.07 (1.00–1.14)
4 HPV subtype	194	1.6	0.31 (0.27–0.35)
5 HPV subtype	66	0.54	0.11 (0.08–0.13)
6 HPV subtype	39	0.32	0.06 (0.05–0.08)
7 HPV subtype	10	0.082	0.02 (0.01–0.03)
8 HPV subtype	5	0.042	0.01 (0.00–0.01)

**Table 3:** The HPV prevalence according to age groups and infection pattern.

**Prevalence and proportion (%)**	**Age group, years**						
	**< 18 (** **n** = 66**)**	**18–29 (** **n** = 7259**)**	**30~39 (** **n** = 20,922**)**	**40~49 (** **n** = 17,913**)**	**50~59 (** **n** = 11,608**)**	**≥ 60 (** **n** = 4268**)**	**Total (** **n** = 62,036**)**
Total	45 (68.18)	1595 (21.97)	3350 (16.01)	2868 (16.01)	2842 (24.48)	1444 (33.83)	12,144 (19.58)
Single HPV	14 (21.21, 31.11)	1024 (14.11, 64.20)	2610 (12.47, 77.91)	2295 (12.81, 80.02)	2074 (17.87, 72.98)	867 (20.31, 60.04)	8884 (14.32, 73.16)
Double HPV	12 (18.18, 26.67)	349 (4.81, 21.88)	580 (2.77, 17.31)	435 (2.43, 15.17)	549 (4.73, 19.32)	368 (8.62, 25.48)	2293 (3.70, 18.88)
Multiple HPV	19 (28.79, 42.22)	222 (3.06, 13.92)	160 (0.76, 4.78)	138 (0.77, 4.81)	219 (1.89, 7.71)	209 (4.90, 14.47)	967 (1.56, 7.96)

**Table 4:** Single and multiple type infection rates of different HPV subtypes.

**HPV subtype**	**Positive, ** **n** ** (%)**	**95% CI for positive (%)**	**Single infection, ** **n** ** (%)**	**Multiple infections, ** **n** ** (%)**
High-risk HPV				
HPV52	2566 (4.14)	3.98–4.29	1646 (2.65)	920 (1.48)
HPV16	1513 (2.44)	2.32–2.56	898 (1.45)	615 (0.99)
HPV53	1472 (2.37)	2.25–2.49	777 (1.25)	695 (1.12)
HPV58	1469 (2.37)	2.25–2.49	810 (1.31)	659 (1.06)
HPV56	929 (1.50)	1.40–1.59	474 (0.76)	455 (0.73)
HPV68	926 (1.49)	1.40–1.49	463 (0.75)	463 (0.75)
HPV51	851 (1.37)	1.28–1.46	390 (0.63)	461 (0.74)
HPV33	634 (1.02)	0.94–1.10	310 (0.50)	324 (0.52)
HPV59	618 (1.00)	0.92–1.07	302 (0.49)	316 (0.51)
HPV18	529 (0.85)	0.78–0.93	271 (0.44)	258 (0.42)
HPV66	396 (0.64)	0.58–0.70	172 (0.28)	224 (0.36)
HPV35	385 (0.62)	0.56–0.68	190 (0.31)	195 (0.31)
HPV39	366 (0.59)	0.53–0.65	226 (0.36)	140 (0.23)
HPV31	348 (0.56)	0.50–0.62	186 (0.30)	162 (0.26)
HPV45	241 (0.39)	0.34–0.44	111 (0.18)	130 (0.21)
HPV73	112 (0.18)	0.15–0.21	40 (0.06)	72 (0.12)
HPV82	67 (0.11)	0.08–0.13	28 (0.05)	39 (0.06)
Low-risk HPV				
HPV81	1347 (2.17)	2.06–2.29	671 (1.08)	676 (1.09)
HPV42	662 (1.07)	0.99–1.15	295 (0.48)	367 (0.59)
HPV43	601 (0.97)	0.89–1.05	258 (0.42)	343 (0.55)
HPV6	459 (0.74)	0.67–0.81	198 (0.32)	261 (0.42)
HPV11	282 (0.45)	0.40–0.51	120 (0.19)	162 (0.26)
HPV83	116 (0.19)	0.15–0.22	48 (0.08)	68 (0.11)

**Table 5:** The prevalence of HPV genotype infection by age group.

**HPV subtype ** **n** ** (%)**	**Age group, years**					
	**< 18 (** **n** = 66**)**	**18–29 (** **n** = 7259**)**	**30~39 (** **n** = 20,922**)**	**40~49 (** **n** = 17,913**)**	**50~59 (** **n** = 11,608**)**	**> 60 (** **n** = 4268**)**
High-risk HPV						
HPV52	11 (16.67)	385 (5.30)	680 (3.25)	579 (3.23)	551 (4.75)	360 (8.43)
HPV16	11 (16.67)	267 (3.68)	411 (1.96)	307 (1.71)	299 (2.58)	218 (5.11)
HPV53	3 (4.55)	176 (2.42)	326 (1.56)	340 (1.90)	407 (3.51)	220 (5.15)
HPV58	10 (15.15)	184 (2.53)	376 (1.80)	316 (1.76)	353 (3.04)	230 (5.39)
HPV56	5 (7.58)	106 (1.46)	183 (0.87)	217 (1.21)	245 (2.11)	173 (4.05)
HPV68	6 (9.09)	126 (1.74)	233 (1.11)	208 (1.16)	217 (1.87)	136 (3.19)
HPV51	8 (12.12)	171 (2.36)	235 (1.12)	158 (0.88)	178 (1.53)	101 (2.37)
HPV33	4 (6.06)	73 (1.01)	140 (0.67)	112 (0.63)	158 (1.36)	147 (3.44)
HPV59	2 (3.03)	117 (1.61)	184 (0.88)	140 (0.78)	126 (1.09)	49 (1.15)
HPV18	4 (6.06)	93 (1.28)	149 (0.71)	115 (0.64)	110 (0.95)	58 (1.36)
HPV66	3 (4.55)	61 (0.84)	94 (0.45)	81 (0.45)	105 (0.90)	52 (1.22)
HPV35	2 (3.03)	46 (0.63)	89 (0.43)	70 (0.39)	110 (0.95)	68 (1.59)
HPV39	2 (3.03)	48 (0.66)	110 (0.53)	103 (0.58)	67 (0.58)	36 (0.84)
HPV31	4 (6.06)	46 (0.63)	87 (0.42)	76 (0.42)	70 (0.60)	65 (1.52)
HPV45	0 (0)	23 (0.32)	66 (0.32)	60 (0.33)	56 (0.48)	36 (0.84)
HPV73	3 (4.55)	24 (0.33)	32 (0.15)	16 (0.09)	24 (0.21)	13 (0.30)
HPV82	0 (0)	10 (0.14)	16 (0.08)	24 (0.13)	42 (0.36)	24 (0.56)
Low-risk HPV						
HPV81	9 (13.64)	162 (2.23)	315 (1.51)	310 (1.73)	374 (3.22)	177 (4.15)
HPV42	7 (10.61)	96 (1.32)	182 (0.87)	121 (0.68)	178 (1.53)	78 (1.83)
HPV43	3 (4.55)	106 (1.46)	166 (0.79)	136 (0.76)	127 (1.09)	63 (1.48)
HPV6	16 (24.24)	110 (1.52)	112 (0.54)	72 (0.40)	100 (0.86)	49 (1.15)
HPV11	13 (19.70)	59 (0.81)	91 (0.43)	45 (0.25)	47 (0.40)	27 (0.63)
HPV83	0 (0)	10 (0.14)	16 (0.08)	24 (0.13)	42 (0.36)	24 (0.56)

**Table 6:** The prevalence of HPV among different age women in GCG and PEG groups.

**Age, years**	**GCG**			**PEG**		
	**Sample size**	**Positive no**	**Prevalence (95% CI, %)**	**Sample size**	**Positive no**	**Prevalence (95% CI, %)**
< 18	66	45	68.18 (56.94–79.42)	0	0	—
18–29	5786	1514	26.17 (25.03–27.30)	1473	81	5.50 (4.33–6.66)
30~39	13,252	2796	21.10 (20.40–21.79)	7670	554	7.22 (6.64–7.80)
40~49	11,000	2376	21.60 (20.83–22.37)	6913	492	7.12 (6.51–7.72)
50~59	7846	2527	32.21 (31.17–33.24)	3762	315	8.37 (7.49–9.26)
≥ 60	3224	1300	40.32 (38.63–42.02)	1044	144	13.79 (11.70–15.88)
Total	41,174	10,558	25.64 (25.22–26.06)	20,862	1586	7.60 (7.24–7.96)

**Table 7:** The distribution of HPV genotypes in GCG and PEG groups.

**HPV subtype**	**GCG**		**PEG**	
	**n**	**Prevalence (95% CI, %)**	**n**	**Prevalence (95% CI, %)**
High-risk HPV				
HPV52	2166	5.26 (5.08–5.45)	400	1.93 (1.75–2.12)
HPV53	1402	3.41 (3.25–3.56)	70	0.34 (0.26–0.42)
HPV16	1375	3.34 (3.19–3.49)	138	0.67 (0.56–0.78)
HPV58	1232	2.99 (2.85–3.13)	237	1.15 (1.00–1.29)
HPV68	844	2.05 (1.93–2.17)	82	0.40 (0.31–0.48)
HPV56	814	1.98 (1.86–2.09)	115	0.56 (0.45–0.66)
HPV51	764	1.86 (1.74–1.97)	87	0.42 (0.33–0.51)
HPV33	586	1.42 (1.32–1.52)	48	0.23 (0.17–0.30)
HPV59	515	1.25 (1.16–1.34)	103	0.50 (0.40–0.59)
HPV18	461	1.12 (1.03–1.21)	68	0.33 (0.25–0.41)
HPV66	374	0.91 (0.83–0.99)	22	0.11 (0.06–0.15)
HPV35	339	0.82 (0.75–0.90)	46	0.22 (0.16–0.29)
HPV31	301	0.73 (0.66–0.80)	47	0.23 (0.16–0.29)
HPV39	222	0.54 (0.48–0.60)	144	0.70 (0.58–0.81)
HPV45	208	0.51 (0.45–0.56)	33	0.16 (0.11–0.21)
HPV73	110	0.27 (0.22–0.31)	2	0.01 (0.00–0.02)
HPV82	64	0.16 (0.12–0.19)	3	0.01 (0.00–0.03)
Low-risk HPV				
HPV81	1301	3.16 (3.01–3.31)	46	0.22 (0.16–0.29)
HPV42	650	1.58 (1.48–1.68)	12	0.06 (0.03–0.09)
HPV43	575	1.40 (1.30–1.49)	26	0.13 (0.08–0.17)
HPV6	409	0.99 (0.91–1.08)	50	0.24 (0.17–0.31)
HPV11	262	0.64 (0.57–0.70)	20	0.10 (0.05–0.14)
HPV83	109	0.26 (0.22–0.31)	7	0.03 (0.01–0.06)

## Data Availability

The data that support the findings of this study are available from the corresponding author upon reasonable request.

## Supporting Information

Supporting Information 1 Data S1: In order to ensure the comparability and accuracy of the reagents used in the laboratory and different brands of reagents in other laboratories, the HPV project of the laboratory regularly participate in the national laboratory quality assessment activity every year. Data S1 presents the results of our laboratory's participation in the 2022 Chinese National Laboratory Inter-laboratory Quality Assessment for the HPV project.

Supporting Information 2 Data S2: The test principle, product performance and result interpretation example of the kit manual, and examples of the actual results.
