# Supplementary material for: Epidemiological Analysis of HPV Infection in Zhangjiagang, Southern Jiangsu Province of China: A Cross-Sectional Study
Source: Int J Microbiol. 2025 Apr 19;2025:5576260. doi: 10.1155/ijm/5576260 (PMC12033064; doi:10.1155/ijm/5576260)
Supplement: Supporting Information 1 — Data S1: In order to ensure the comparability and accuracy of the reagents used in the laboratory and different brands of reagents in other laboratories, the HPV project of the laboratory regularly participate in the national laboratory quality assessment activity every year. Data S1 presents the results of our laboratory's participation in the 2022 Chinese National Laboratory Inter-laboratory Quality Assessment for the HPV project. [file 5576260.f1.pdf]

2022 National External Quality Assessment
Report for Clinical Laboratories

Unit (Laboratory) Code: 121257

Unit (Laboratory) Name: Zhangjiagang First People's Hospital, Suzhou City, Jiangsu Province

Program Name: Human Papillomavirus (HPV) Genotyping

Program Code: NCCL-I-13

Assessment Round: 2nd

Responsible Department: Clinical Immunology Laboratory

Contact Phone: 010-58115053

Mailing Address:: National Center for Clinical Laboratories, National Health Commission
No. 1 Donghua University Road, Beijing, China
Postal Code: 100730
Phone: 010-58115055, 65273025, 58115065
Fax: 010-65273025
Email: EQ4@nccl.org.cn

National Center for Clinical Laboratories, National Health Commission

Laboratory Name: Zhangjiagang First People’s Hospital, Suzhou City, Jiangsu Province
Department: Laboratory Medicine
Laboratory Code: 121257 **Testing Date**: 2022/9/7 **Round**: 2nd **Statistical Date**: 2022/9/29

Sample ID	Expected Result	Your Lab Result	Pass/Fail	National Correct Rate (%)
202221	HPV low-risk type、HPV-11	HPV-11	Pass	98.75
202222	HPV high-risk type、HPV-16、HPV-18	HPV-16、HPV-18	Pass	98.81
202223	Negative	Negative	Pass	99.62
202224	HPV high-risk type、HPV-52	HPV-52	Pass	99.00
202225	HPV high-risk type、HPV-33	HPV-33	Pass	99.06

Total Score 100

202221 Sample results

Mutation Type	Expected Result	Your Lab Result	Pass/Fail	National Correct Rate (%)
HPV high-risk type	N	Not detectet		100
HPV-16	N	N	Pass	100
HPV-18	N	N	Pass	100
HPV-31	N	N	Pass	100
HPV-33	N	N	Pass	99.94
HPV-35	N	N	Pass	100
HPV-39	N	N	Pass	100
HPV-42	N	N	Pass	100
HPV-43	N	N	Pass	100
HPV-44	N	N	Pass	100
HPV-45	N	N	Pass	99.94
HPV-51	N	N	Pass	100
HPV-52	N	N	Pass	100
HPV-53	N	N	Pass	100
HPV-56	N	N	Pass	100
HPV-58	N	N	Pass	100

HPV-59	N	N	Pass	100
HPV-66	N	N	Pass	100
HPV-68	N	N	Pass	100
HPV-81	N	N	Pass	100
HPV low-risk type	P	Not detectet		91.18
HPV-6	N	N	Pass	99.86
HPV-11	P	P	Pass	99.15

202222 Sample results

Mutation Type	Expected Result	Your Lab Result	Pass/Fail	National Correct Rate (%)
HPV high-risk type	P	Not detectet		94.37
HPV-16	P	P	Pass	99.75
HPV-18	P	P	Pass	99.49
HPV-31	N	N	Pass	99.87
HPV-33	N	N	Pass	100
HPV-35	N	N	Pass	100
HPV-39	N	N	Pass	100
HPV-42	N	N	Pass	100
HPV-43	N	N	Pass	100
HPV-44	N	N	Pass	100
HPV-45	N	N	Pass	99.94
HPV-51	N	N	Pass	100
HPV-52	N	N	Pass	100
HPV-53	N	N	Pass	100
HPV-56	N	N	Pass	100
HPV-58	N	N	Pass	100
HPV-59	N	N	Pass	99.87
HPV-66	N	N	Pass	100
HPV-68	N	N	Pass	100
HPV-81	N	N	Pass	100
HPV low-risk type	N	Not detectet		98.53
HPV-6	N	N	Pass	100
HPV-11	N	N	Pass	99.86

National Center for Clinical Laboratories, National Health Commission
2022 National HPV Genotyping External Quality Assessment Statistical Results

[illegible]gjiagang First People’s Hospital, Suzhou City, Jiangsu Province

Department: Laboratory Medicine

Laboratory Code: 121257 **Testing Date**: 2022/9/7 **Round**: 2nd **Statistical Date**: 2022/9/29

202223 Sample results

Mutation Type	Expected Result	Your Lab Result	Pass/Fail	National Correct Rate (%)
HPV high-risk type	N	Not detectet		98.59
HPV-16	N	N	Pass	99.94
HPV-18	N	N	Pass	99.68
HPV-31	N	N	Pass	100
HPV-33	N	N	Pass	100
HPV-35	N	N	Pass	100
HPV-39	N	N	Pass	100
HPV-42	N	N	Pass	100
HPV-43	N	N	Pass	100
HPV-44	N	N	Pass	100
HPV-45	N	N	Pass	100
HPV-51	N	N	Pass	100
HPV-52	N	N	Pass	100
HPV-53	N	N	Pass	100
HPV-56	N	N	Pass	100
HPV-58	N	N	Pass	100
HPV-59	N	N	Pass	100
HPV-66	N	N	Pass	100
HPV-68	N	N	Pass	100
HPV-81	N	N	Pass	100
HPV low-risk type	N	Not detectet		100
HPV-6	N	N	Pass	100
HPV-11	N	N	Pass	100

202224 Sample results

Mutation Type	Expected Result	Your Lab Result	Pass/Fail	National Correct Rate (%)
HPV high-risk type	P	Not detectet		92.96
HPV-16	N	N	Pass	99.62
HPV-18	N	N	Pass	100
HPV-31	N	N	Pass	100
HPV-33	N	N	Pass	100
HPV-35	N	N	Pass	100
HPV-39	N	N	Pass	100

HPV-42	N	N	Pass	100
HPV-43	N	N	Pass	100
HPV-44	N	N	Pass	100
HPV-45	N	N	Pass	100
HPV-51	N	N	Pass	100
HPV-52	P	P	Pass	99.68
HPV-53	N	N	Pass	100
HPV-56	N	N	Pass	100
HPV-58	N	N	Pass	100
HPV-59	N	N	Pass	99.94
HPV-66	N	N	Pass	100
HPV-68	N	N	Pass	100
HPV-81	N	N	Pass	100
HPV low-risk type	N	Not detectet		98.53
HPV-6	N	N	Pass	100
HPV-11	N	N	Pass	100

202225 Sample results

Mutation Type	Expected Result	Your Lab Result	Pass/Fail	National Correct Rate (%)
HPV high-risk type	P	Not detectet		94.37
HPV-16	N	N	Pass	100
HPV-18	N	N	Pass	100
HPV-31	N	N	Pass	99.94
HPV-33	P	P	Pass	99.49
HPV-35	N	N	Pass	99.94
HPV-39	N	N	Pass	100
HPV-42	N	N	Pass	100
HPV-43	N	N	Pass	100
HPV-44	N	N	Pass	100
HPV-45	N	N	Pass	100
HPV-51	N	N	Pass	100
HPV-52	N	N	Pass	100
HPV-53	N	N	Pass	99.79
HPV-56	N	N	Pass	100
HPV-58	N	N	Pass	99.94

Laboratory Name: Zhangjiagang First People's Hospital, Suzhou City, Jiangsu Province **Department**: Laboratory Medicine
Laboratory Code: 121257 **Testing Date**: 2022/9/7 **Round**: 2nd **Statistical Date**: 2022/9/29

HPV-59	N	N	Pass	100
HPV-66	N	N	Pass	100
HPV-68	N	N	Pass	99.94
HPV-81	N	N	Pass	100
HPV low-risk type	N	Not detectet		98.53
HPV-6	N	N	Pass	100
HPV-11	N	N	Pass	100
