# Supplementary material for: Epidemiological Analysis of HPV Infection in Zhangjiagang, Southern Jiangsu Province of China: A Cross-Sectional Study
Source: Int J Microbiol. 2025 Apr 19;2025:5576260. doi: 10.1155/ijm/5576260 (PMC12033064; doi:10.1155/ijm/5576260)

Supplementary material 2: Test Principle and Performance of the kit (excerpt from the kit manual) and Result image example

[Name of Test Kit]: Human papillomavirus Genotyping (Type 23) Test Kit (PCR-Reverse dot hybridization)

[Test principle of kit]

This kit uses a combination of PCR in vitro amplification and DNA reverse dot hybridization to detect HPV genotyping. By designing specific primers based on the genetic characteristics of HPV, the target fragments of 23 HPV genotypes can be amplified, and then the amplified products are hybridized with the typing probes fixed on the membrane strip, including 17 high-risk types and 6 low-risk types, and the infection of these HPV genotypes can be judged according to the presence or non-presence of hybridization signals.

[Product performance index]

1. Analytical sensitivity

Sensitivity analysis was performed on 23 genotypes, 3 samples per genotype, each containing 4 concentration gradients. The minimum detectable limit of human papillomavirus (HPV) pathogens was determined to be 1.0×10^4copies/mL.

2. Measurement accuracy

The product was used for accuracy analysis of 23 genotypes, 3 samples for each genotype, and each sample contained 3 concentration gradients for high, middle and low levels. The positive coincidence rate was 100%. The samples infected by other common pathogens (Chlamydia trachomatis, Mycoplasma hominis, Candida albicans, Trichomonas vaginalis, Treponema pallidum, Streptococcus pyogenes and herpes simplex virus type II) were analyzed accurately, and the negative coincidence rate was 100%.

3. Analyze specificity

Cross-reactivity analysis was performed on other 7 common STD pathogens (Chlamydia trachomatis, Mycoplasma hominis, Candida albicans, Trichomonas vaginalis, Treponema pallidum, Streptococcus pyogenes and herpes simplex virus type II) and other 7 HPV genotypes (HPV26, 44, 54, 55, 61, 67 and 70) outside the

detection scope of this product. Results There was no cross reaction.

4. Inaccuracy and inaccuracy between batches

This product assesses clinical samples of two concentration gradients, and the coefficient of variation (CV) of inaccuracy and inaccuracy between batches is <5%.

[Result image example]

1.Result display example from kit instructions(HPV11+, HPV16+)

HPV11

6	11●	16	18	31	33	35	39
42	43	45	51	52	53	56	58
59	66	68	73	81	82	83	IC●

HPV16

6	11	16●	18	31	33	35	39
42	43	45	51	52	53	56	58
59	66	68	73	81	82	83	IC●

2.Result display example from actual picture

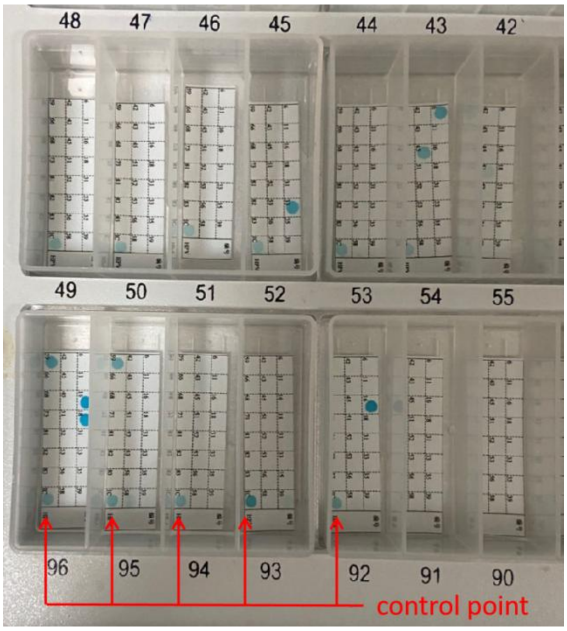

Supplement: Supporting Information 2 — Data S2: The test principle, product performance and result interpretation example of the kit manual, and examples of the actual results. [file 5576260.f2.pdf]
